# Molecular basis for loss of virulence in *Magnaporthe oryzae* strain AM16

**DOI:** 10.3389/fpls.2024.1484214

**Published:** 2024-12-06

**Authors:** Jiahui Deng, Ziya Zhang, Xingli Wang, Yongni Cao, Huichuan Huang, Mo Wang, Qiong Luo

**Affiliations:** State Key Laboratory for Conservation and Utilization of Bio-Resources in Yunnan, Yunnan Agricultural University, Kunming, China

**Keywords:** *Magnaporthe oryzae*, AM16 strain, virulence variation, Pmk1, Mac1

## Abstract

The rapid virulence variation of *Magnaporthe oryzae* (*M. oryzae*) to rice is a big challenge for rice blast control. Even though many studies have been done by scientists all over the world, the mechanism of virulence variation in *M. oryzae* remains elusive. AM16, an avirulent *M. oryzae* strain reported in our previous study, provides an excellent entry point to explore the mechanism of virulence variation in *M. oryzae*. In this study, we found that the *Pmk1* and *Mac1* had specific mutations in strain AM16. The AM16 strains overexpressing *Pmk1* ^Guy11^ or (and) *Mac1* ^Guy11^ allele from strain Guy11 displayed significantly increasing conidiation, functional appressorium formation, and restoring pathogenicity to rice. Moreover, we observed that the strains overexpressing *Mac1* ^Guy11^ had stronger conidia forming capacity than that of the strains overexpressing *Pmk1* ^Guy11^, while the appressorium formation rate of strains overexpressing *Pmk1* ^Guy11^ was similar to that of strains overexpressing *Pmk1* ^Guy11^-*Mac1* ^Guy11^, much higher than that of the strains overexpressing *Mac1* ^Guy11^. Taken together, our results reveal that the natural mutation of *Pmk1* and *Mac1* genes are important, but not the sole cause, for the loss of virulence in strain AM16. The functional difference between Pmk1 and Mac1 in the growth and development of *M. oryzae* was first discovered, providing new insight into the pathogenic mechanism of *M. oryzae*.

## 1 Introduction

Rice, as one of the major cereal crops, feeds more than half of the world’s population and contributes significantly to global food security (Shi et al., 2023). Rice blast, caused by *M. oryzae*, is responsible for significant reductions in rice yield annually (Dagdas et al., 2012; Li et al., 2020). Controlling the incidence of rice blast is an essential for stabilizing global food security. Two main measures are used for controlling rice blast in rice production: planting disease-resistant varieties and applying chemical pesticides (Younas et al., 2023). Breeding and using resistant varieties is considered to be the most cost-effective and environmentally friendly strategy to control rice blast (Liu et al., 2021; Gao et al., 2023). However, the genetic resistance of a rice cultivar is often broken down in a few years due to the emergence of new virulent pathotypes of *M. oryzae* in field (Damchuay et al., 2020; Kaur et al., 2021). Therefore, an understanding of virulence variation mechanisms of rice blast fungus is fundamental to developing resistance rice varieties for disease control.

*M. oryzae* infection begins with the germination of conidia on the leaf surface, and the tip of the germ tube senses and responds to various physical signals from the host, forming dome-shaped infection structure called appressorium. Under the deposition of melanin and chitin, the appressorium forms an infection peg and invades the host cell. This leads to the formation of primary infection hyphae, which elongate and differentiate within the host cell (Osés-Ruiz et al., 2017; Ryder et al., 2019; Gupta et al., 2021). Then, the secondary infection hyphae are produced, expanding through plasmodesmata into neighboring cells. Eventually, this process leads to the host cell’s death and the host disease’s development (Kankanala et al., 2007; Deng and Naqvi, 2019). The conidiation and functional appressorium are crucial for successful infection of *M. oryzae*.

The physical signal perception of the host surface, conidial germination, appressorium formation, penetration of host epidermal cells and growth of infected hyphae are regulated by G protein/cAMP-dependent signal transduction pathway, Pmk1-MAP kinase and TOR kinase signaling pathways (Dean, 1997; Zhang et al., 2011; He et al., 2018; Wang et al., 2021). Upon conidia germination, the G-protein coupled receptor Pth11 senses hydrophobic surface, the key activators and regulators of G-protein/cAMP (Pth11, MoSho1, G-proteins, RGS1 and Mac1) are internalized to the endosomal compartments. Pth11 interacts with the G-protein complex leading to the accumulation of cAMP and subsequently PKA activity (Fernandez and Orth, 2018). Two forms of PKA, CpkA and Cpk2, have overlapping functions in the cAMP cascade (Li et al., 2012), which is essential for normal appressorium development and pathogenicity (Kou et al., 2017). The adenylate cyclase Mac1 catalyzes cAMP synthesis in *M. oryzae*, its deletion leads to defects in hyphal growth, conidiation, appressorium formation, and pathogenicity (Choi and Dean, 1997; Adachi and Hamer, 1998; Wilson and Talbot, 2009).

The Pmk1 MAP kinase pathway regulates late stages of appressorium formation, penetration and invasive growth (Zhang et al., 2011; Li et al., 2012; Zhang et al., 2016; Sakulkoo et al., 2018; Zhang et al., 2019). Loss in Pmk1 function results in an appressorium-defective mutant that is nonpathogenic. Upstream to Pmk1, Mst7, Mst11 and PoRal2 interact with Mst50 to activate the Pmk1 MAPK cascade, the deletion of any of these genes upstream of Pmk1 results in impairments of appressorium formation and plant infection (Zhao et al., 2005; Park et al., 2006; Fernandez and Orth, 2018; Qu et al., 2021). While, artificial activation of the cAMP-PKA and Pmk1 MAPK pathways by dominant expression of the *RAS2* ^G18V^ allele resulted in the formation of appressorium on hydrophilic surfaces (Zhou et al., 2014).

Besides, Marroquin and Wilson have demonstrated that activation of the target of rapamycin (TOR) signaling pathway, in response to the accumulation of intracellular glutamine, triggers the inhibition of appressorium development. The GATA transcription factor, Asd4, regulates intracellular glutamine levels and promotes appressorium formation. Δasd4 mutant strains are unable to develop an appressorium on an inductive surface, and they contain high levels of glutamine (Marroquin-Guzman and Wilson, 2015). Interestingly, inhibition of the TOR pathway by rapamycin restores appressorium formation in both *asd4* and *cpka* mutants but not in the MAP kinase *pmk1* mutant. Therefore, TOR inactivation requires a functional Asd4 to regulate intracellular glutamine levels and develop an appressorium in *M. oryzae* (Marroquin-Guzman and Wilson, 2015; Fernandez and Orth, 2018).

The *M. oryzae* strain AM16 was avirulent to all rice varieties tested in our study, including the most susceptible rice variety Lijiang Xintuan Heigu (LTH) (Wang et al., 2022). Preliminary experiments found that the AM16 strain had defects in conidiation and appressorium formation. In this study, we identified two genes *Pmk1* and *Mac1* with specific mutations in the AM16 strain by analyzing 13 key genes of the Cyclic AMP, TOR Kinase, and Pmk1 MAP Kinase Pathways and revealed that the mutations of *Pmk1* and *Mac1* are the important cause for the loss of pathogenicity in the strain AM16. Besides, our study first found the functional differences between Pmk1 and Mac1 in the growth and development of *M. oryzae*.

## 2 Materials and methods

### 2.1 Rice materials and growth conditions

Jiangnan Xiangnuo (JNXN), Nipponbare (Nip) and Lijiang Xintuan heigu (LTH) are three susceptible rice varieties used in this study, preserved in our laboratory. Healthy and mature seeds were sterilized with 75% ethanol for 40 seconds, then 20% sodium hypochlorite for 40 minutes, rinsed with pure water, soaked in water at 37 °C for 24 h. After the seeds are fully absorbed, and placed in a 32 °C light incubator to germination. The germinated seeds were planted in 96-well PCR plates with the bottom of the tube cut off, and grown in a nutrient solution at room temperature, relative humidity (70 ± 10) % with a cycle of 12 h light and 12 h dark period. The nutrient solution formulation reference (IRRI, 1996), and changed once a week.

### 2.2 Fungal isolates, culture conditions and trait observation

The pathogenic strain Guy11 was kindly provided by Prof. Mo Wang (Yunnan Agricultural University). The pathogenic strain A7203-8 was isolated and preserved by our laboratory. The avirulent strain AM16, the original strain was provided by the Sichuan Academy of Agricultural Sciences.

*M. oryzae* strains were activated on potato sucrose agar (PSA) medium (20 g sucrose,18 g agar, 200 g potato collected the liquid filter after boiling, add distilled water up to 1000 mL) for 5 days at 28 °C, 30 mycelial plugs (1 mm in diameter) were transferred into conical flask with 100 ml potato sucrose broth (PSB) medium (20 g sucrose, 200 g potato collected the liquid filter after boiling, add distilled water up to 1000 mL), 180 rpm/min, at 28 °C for 4 days, 700 μL of mycelium solution were spread on the oat-tomato agar (OTA) plate (40 g oatmeal collected the liquid filter after boiling, 200 ml fresh tomato juice, 18 g agar, add distilled water up to 1000 mL) for 4 days at 28 °C under natural light, then conidial spores were collected via flooding of the fungal agar cultures with sterile distilled water, the spore concentration was adjusted to 2x10^5^ conidia/mL. All the media were autoclaved at 121 °C for 20 min and dispensed into petri dishes at 30 ml per plate.

For trait observation, *M. oryzae* strains were activated on potato sucrose agar (PSA) medium for 3 days at 28 °C, mycelial plug (1 mm in diameter) was transferred to fresh PSA medium for 9 days at 28 °C. The colony morphology, color, and diameter were investigated. Three replicates.

### 2.3 Conidiation assay

700 μL of mycelium solution were spread on the oat-tomato agar (OTA) plate for 4 days at 28 °C under natural light, then conidial spores were collected via flooding with 4 mL sterile distilled water for each petri dish and filtered through a Miracloth filter. The number of spores was counted with a Hemocytometer Blood Count Plate, under a 10x microscope (Olympus fluorescence microscope BX53), three biological replicates, six fields were counted per replicate. Statistical analysis and graphing of data were performed using GraphPad.

### 2.4 Mycelial RNA and DNA extraction

*M. oryzae* Fungal hyphae were harvested from 4-day-old liquid PSB cultures, blotted on sterile filter paper for total RNA isolation or genome DNA extraction. DNA was extracted using the conventional CTAB method. Total RNA was isolated with TaKaRa MiniBEST Plant RNA Extraction Kit (Code No.9769). The RNA quality was qualitatively determined by 2% agarose gel electrophoresis, and the RNA concentration was determined by NanoDrop 2000 UVev is Spectrophotometer.

### 2.5 Gene cloning and analysis

Specific PCR primers were designed based on 13 pathogenic genes in the Pmk1-MAPK signaling pathway and cAMP signaling pathway (**Supplementary Table S1**). Relevant alleles were cloned from strain AM16, pathogenic strains Guy11 and A7203-8 by PCR amplification using High-fidelity enzyme KOD. The PCR products were observed and photographed using a UV gel imaging system. The PCR products, confirmed by electrophoresis, were sent for sequencing by Qingke Life Science Technology Co, Ltd. Sequence alignment was performed using SnapGene software. Gene sequence changes were analyzed using GeneDoc software.

### 2.6 Protoplast transformation and transformants screening of AM16 strain

A 1071 bp cDNA fragment of *Pmk1* allele (*Pmk1* ^Guy11^) and a 6484 bp cDNA fragment of *Mac1* allele (*Mac1* ^Guy11^) were amplified from pathogenic *M. oryzae* strain Guy11 using the specific PCR primers (**Supplementary Table S1**) and cloned into the PCB1532 vector the Seamless Assembly Cloning Kit to generate the overexpression construct PCB1532-*Pmk1* (*Pmk1*-ox) and PCB1532-*Mac1* (*Mac1*-ox), respectively. After sequence verification, the final construct of *Pmk1*-ox and *Mac1*-ox were transformed into AM16 strain via PEG-mediated protoplast transformation, respectively. Transformants were selected on Potato Sucrose Agar (PSA) medium containing SUR (Chlorimuron ethyl 50 mg/ml). The SUR-resistant transformants were verified PCR using SUR-F/SUR-R primers, *Pmk1*-specific primers, and *Mac1*-specific primers (**Supplementary Table S1**).

LB Medium (For cultivation of *E. coli*): (1 L) Tryptone 10 g, Yeast extract 5 g, NaCl 5 g, pH adjusted to 7.0. Solid LB medium requires the addition of Agar 15 g.

### 2.7 Conidiation germination and appressorium formation on hydrophobic surface

Spores were collected via flooding of the fungal agar cultures with 4 mL sterile water of each petri dish and filtered through a Miracloth filter. The concentration of conidial suspension was adjusted to was adjusted to 1.0×10^5^ conidia/mL. 40 μL of the conidial suspension was dropped onto hydrophobic Microscope Cover Glass (fisherbrand, Cat. No. 12541012, China) surface, placed in a dark incubation chamber with humidity control at 28 °C for 24 hours. Conidial germination was counted at 3, 8, 12, and 24 hours, appressorium formation was counted at 12, and 24 hours. A total of 100 conidia were counted for each treatment, three biological replicates. The conidial germination rate and appressorium formation rate were calculated. Statistical analysis and graphing of data were performed using GraphPad. The statistically significant difference was indicated by * (P<0.05) and ** (P<0.01) or different lower-case letters with Student’s t-test (pairwise comparison).

### 2.8 Appressoria formation and penetration

Preparation of conidial suspension following the method described in 2.7. Fresh onion inner epidermis measuring 3 cm in length and 2 cm in width was washed with sterile water to remove impurities. The hydrophobic surface of the epidermis was placed on coverslip facing upwards, 40 μL conidial suspension was dropped onto the hydrophobic surface. Then the slide was placed in a dark incubation chamber at 28 °C with humidity control. The penetration of appressorium into onion epidermal cells was observed at 12, 24, and 36 hours. 20 conidia were counted for each treatment, three biological replicates. Statistical analysis and graphing of data were performed using GraphPad. The statistically significant difference was indicated by * (P<0.05) and ** (P<0.01) or different lower-case letters with Student’s t-test (pairwise comparison). The penetration rate was calculated and subjected to analysis.

### 2.9 Pathogen infection assay and disease evaluation

Preparation of conidial suspension: Conidia were collected by the same method 2.3. The concentration of conidial suspension was adjusted to 2.0×10^5^ conidia/mL, added 4% gelatin and shake well.

Spray inoculation: Conidial suspension was evenly sprayed onto the leaves of three- to four-leaf rice seedlings using a sprayer until small droplets were visible on the leaves. Rice plants were cultured in a dark incubator at 25 °C to 28 °C, relative humidity 90% for 24 hours, subsequently transferred to a moist greenhouse with a cycle of 12 h light and 12 h dark at 25 °C to 28 °C. Disease evaluation was performed 5 days post inoculation (dpi), and the lesion types on rice leaves were observed and scored 0 (resistant) to 9 (susceptible) according to a standard of International Rice Research Institute’s (IRRI, 1996).

Punch inoculation of vitro leaves: 6 cm fragments of rice leaves were cut from the same part of tillering stage rice plants, and two evenly spaced wounds were punctured on the leaves using a needle. The leaves were then soaked in water containing 1 mg/L 6-BA at pH 7.0, and 10 µl conidia suspension (1 × 10^5^ conidia/ml) was dropped on the leaf wound site. At 25 °C to 28 °C,shade culture for 24 hours, then natural light conditions. The disease lesions were pictured 6 dpi, and the lesion length was measured.

Punch inoculation of vivo leaves: The leaves of rice seedling used for holes punched inoculation with *M. oryzae* according to the method of Zhong (Zhong et al., 2018). The disease lesions were pictured 7 dpi. The diseased leaves were sampled for disease evaluation and relative fungal biomass quantification using a DNA-based quantitative PCR method as described by Park (Park et al., 2012).

### 2.10 Gene expression analysis

gDNA Remover TOYOBO reverse transcription kit (FSQ-301) was used to synthesize the first strand of cDNA, and RT-qPCR was performed using GoTaq qPCR Master Mix kit. PCR reactions of 10 μL contained qPCR Mix 5 μL, the forward primer and reverse primer were 0.2 μL each (10 μmol/L), cDNA template 5 ng. Amplification was performed in Bio-Rad CFX96 Real-Time System coupled with a C1000 Thermal Cycler (Bio-Rad, Hercules, CA, USA) at 95 °C for 5 min; 40 cycles of 95 °C for 15 s, 60 °C for 1 min. The reference gene, Ubiquitin, was used for the normalization of the target genes from the RT-qPCR results, 2^-ΔΔCt^ method was used to calculate the relative expression level with three-technique repeats. The primers were synthesized by Kunming Qingke Biological Company (**Supplementary Table S1**).

### 2.11 Western blot assay

Samples of *M. oryzae* Fungal hyphae, harvested from 4-day-old liquid PSB cultures and blotted on sterile filter paper, were frozen and ground in liquid nitrogen and total proteins were extracted with protein extraction buffer (50 mM Tris-HCl pH 7.5, 150 mM NaCl, 0.5 mM EDTA, 1 mM PMSF, 0.5%[v/v] IGEPAL CA-630, 2 μM NaF, 2 μM Na3VO4 and protease inhibitor cocktail). After centrifuging at 18,000 g for 15 min at 4°C, the supernatants were mixed with 5x SDS loading buffer and boiled at 95°C for 5 min. The specific extraction method refers to Zheng et al. (2015). Protein samples were separated by SDS-PAGE gel. Immunoblotting was performed by incubation PVDF membrane (Merck Millipore) with an anti-Pmk1 antibody [Anti-MAP Kinase, Activated (Diphosphorylated ERK-1&2) Antibody (Clone# MAPK-YT)] (Boster Biological Technology, Wuhan, China. Catalog# BM1595) in 1% nonfat-dried milk in TBST overnight, followed by incubation with anti-mouse-horseradish peroxidase secondary antibody (Abbkine, cat# A21010) for 1 h. The chemiluminescence signal was detected using SuperKine™ West Femto Maximum Sensitivity Substrate (Abbkine, cat# BMU102).

### 2.12 Determination of intracellular cAMP level and influence of exogenous cAMP on *M. oryzae* development

The mycelium of strains Guy11, AM16 and *Mac1*-ox-1-1 were collected from 4-day-old liquid PSB cultures and blotted on sterile filter paper. 10 mg of mycelium was mixed with 1.8 mL PBS, crushed by ultrasound (ice bath, 20% power or 200 W, ultrasonic 3 s, 10 s interval, repeated 30 times), centrifuged at 8000 g at 4°C for 10min. The supernatant was used for the measurement of cAMP according to the manufacturer’s instructions. cAMP was detected using a commercial enzyme-linked immunosorbent ELISA kit (Meimian Industrial Co., Ltd., Jiangsu, China). Four-technique repeats biological replicates.

To determine the effect of exogenous cAMP on conidiation, conidia germination and appressorium formation of AM16, the sporulation medium (OTA plate) containing 5 mM exogenous cAMP used for the observation of conidia germination and appressorium formation on hydrophobic surface, as well as the conidial suspensions (1 x 10^5^ conidia/mL) with 5 mM exogenous cAMP for the observation of conidia germination and appressorium formation on hydrophobic surface. Conidia were counted by the same method 2.3. Conidial germination was counted at 3 and 8 hours, appressorium formation was counted at 12, and 24 hours. A total of 100 conidia were counted for each treatment, three biological replicates. The conidial germination rate and appressorium formation rate were calculated. Statistical analysis and graphing of data were performed using GraphPad. The statistically significant difference was indicated by * (P<0.05) and ** (P<0.01).

## 3 Results

### 3.1 Specific mutations of *Pmk1* and *Mac1* genes were identified in AM16 strain

**Figure 1:**
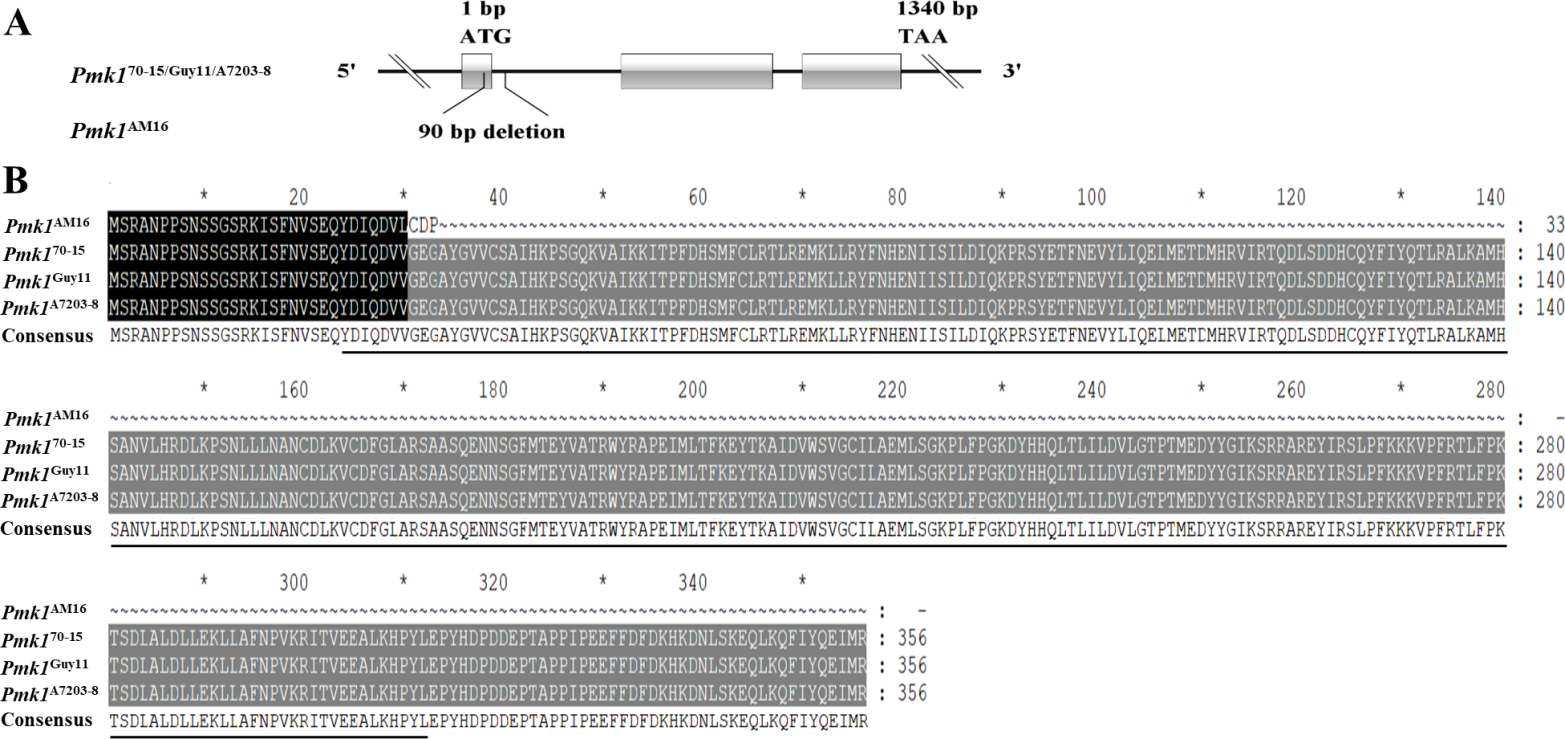
Identification of the *Pmk1* gene. **(A)** The structure of *Pmk1* gene and mutation in the strain AM16. **(B)** Alignment of amino acid sequences. Black underlining indicates the S _ TKC domain; the black shadow represents the same sequence, and the grey shadow represents the mutation sequence. The CDS sequence of *Pmk1* ^70-15^ gene was obtained from NCBI. Gene ID: 2680463. "*" represents a separator inserted every 20 bases.

The specific PCR primers were designed based on the sequences of 13 genes involvement in the cAMP-PKA, Pmk1-MAPK and TOR pathways (**Supplementary Table S1**). Alleles were amplified from the avirulent strain AM16, pathogenic strains Guy11 and A7203-8 by PCR using genomic DNA as templates, respectively. Comparative analysis of 13 gene sequences revealed that 11 alleles (*MoCAP, MoVps39, MoArk1, MoEnd3, MoSfl1, MoSho1, Mst11, Mst50, Mst7, Pth11, CpkA*) were identical sequence among strains AM16, Guy11 and A7203-8. Compared with Guy11 and A7203-8 isolates, the *Pmk1* gene from AM16 strain has 80 bp deletion (862 to 941), including 29 bp in exon 1 and 51 bp in intron 1 (**Figure 1**). Further, we found that the cDNA sequence of *Pmk1* gene from strain AM16, compared with strains Guy11 and A7203-8, retained 85 bp sequence of intron 2 in addition to 29 bp deletion of exon 1, resulting in encoding a truncated protein consisting of 33 amino acids (**Figure 1**; **Supplementary Figure S1**). Pan-genomic analysis of 312 *M. oryzae* isolates showed that the mutations of *Pmk1* gene are unique to strain AM16.

**Figure 2:**
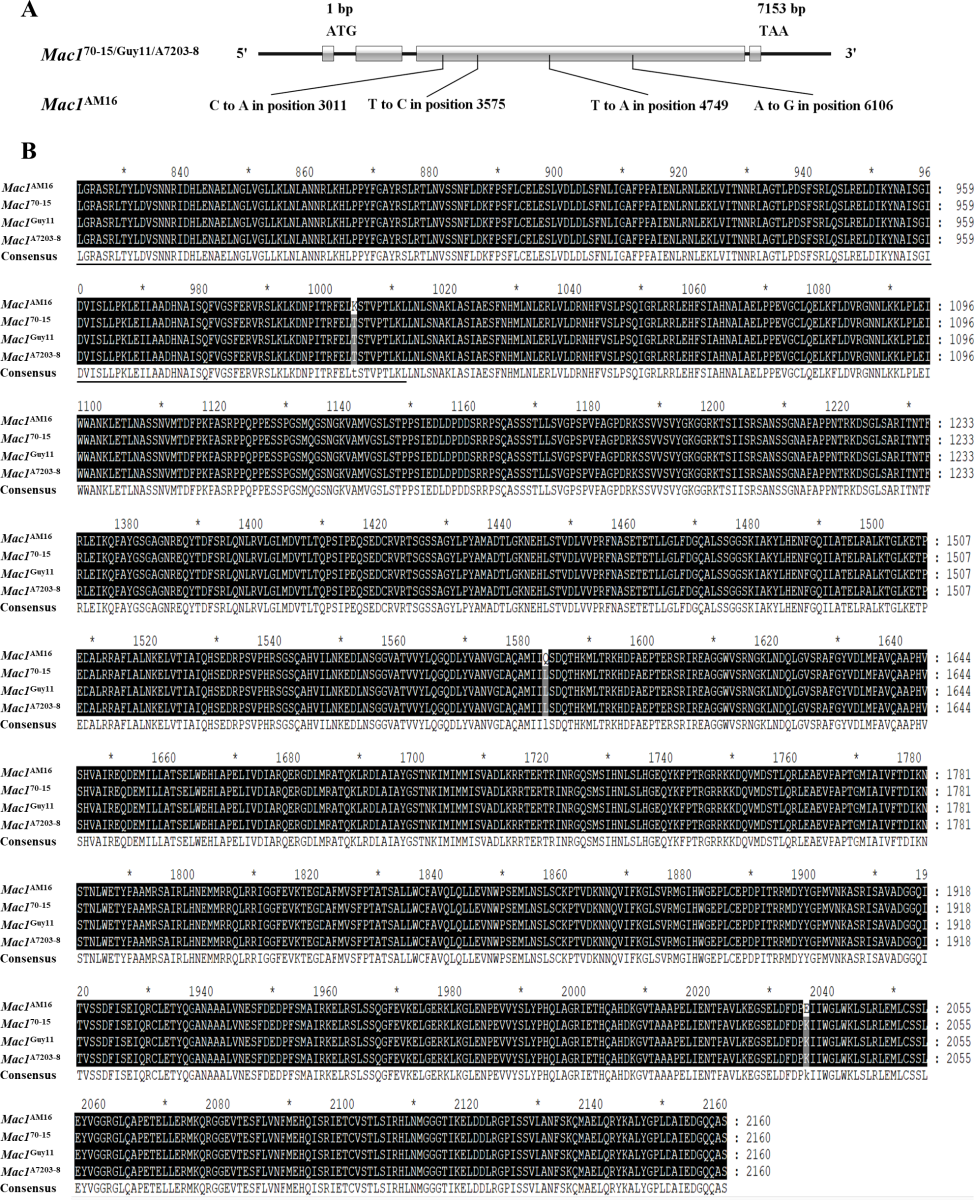
Identification of the *Mac1* gene. **(A)** The structure of *Mac1* gene and mutation in the strain AM16. **(B)** Alignment of amino acid sequences. Black underlining indicates the PLN00113 domain. Black shading indicates identical sequences and gray shading indicates the mutation sites. The CDS sequence of *Mac1* ^70-15^ gene was obtained from NCBI. Gene ID: 2680942. "*" represents a separator inserted every 20 bases.

Four nucleotide substitution mutations leading to non-synonymous substitutions of three amino acids (T1004K, L1583Q and K2036E) were identified in *Mac1* gene from strain AM16, among which T1004K is located in the PLN00113 conserved domain compared to that in strains Guy11 and A7203-8 (**Figure 2**; **Supplementary Figure S2**), and the mutation frequency was about 20% in 312 strains.

### 3.2 *Mac1* and *Pmk1* mutations significantly reduced conidiation in strain AM16

In order to assess the involvement of *Pmk1* and *Mac1* gene mutations in loss of virulence in AM16 strain, we generated the overexpression constructs *Pmk1*-ox and *Mac1*-ox, were transformed into the strain AM16 via PEG-mediated protoplast transformation, respectively. The positive transformants *Pmk1-*ox-9-3, *Mac1-*ox*-*1-1 and *Pmk1-Mac1-*ox-2-2 were selected (**Supplementary Figures S3**-**S5**), and used for the investigations of morphology (colony shape, color, hyphal growth), conidiation, conidial germination, appressorium formation, and pathogenicity.

**Figure 3:**
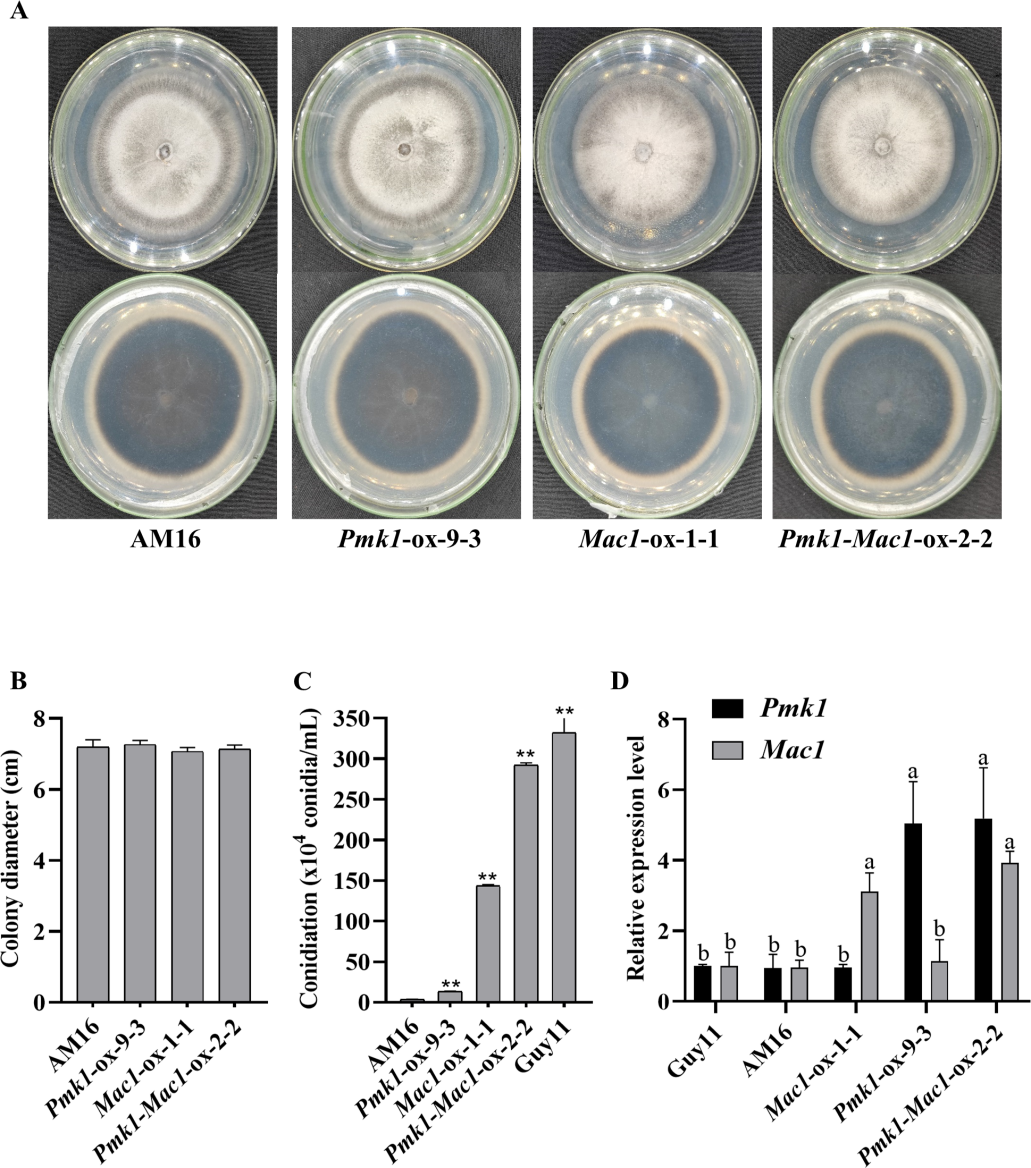
Culture characteristics and conidiation. **(A)** Strain colony morphology. **(B)** Colony diameter (9 dpi). **(C)** Number of conidia. Error bars represent mean ± SD (n =3). Asterisks indicate significant differences from AM16 strain according to Student’s t-test (** p < 0.01). **(D)** The relative expression level of *Pmk1* and *Mac1* genes in five strains. Data were analyzed using ANOVA (P < 0.05) and Duncan’s test. Error bars represent the SD of three replicates (n = 3). Different letters above the bars indicate significant differences at p<0.05.

Although the colony morphology of the transformed strains *Pmk1-*ox-9-3, *Mac1-*ox*-*1-1 and *Pmk1-Mac1*-ox-2-2 was similar to that of the strain AM16 (**Figures 3A, B**), their conidiation competence on OTA plate was significantly different (**Figure 3C**; **Supplementary Figure S6**). Even though overexpressing *Pmk1* or *Mac1* or both did not restore the conidiation of AM16 to the level of virulent strain Guy11, compared with strain AM16, the number of spores produced by strains *Pmk1-*ox-9-3, *Mac1-*ox*-*1-1 and *Pmk1-Mac1-*ox-2-2 increased by 3.9, 41.7 and 84.9 times, respectively (**Figure 3C**; **Supplementary Figure S6**), which was consistent with the expression levels of *Pmk1* or (and) *Mac1* in strains *Pmk1-*ox-9-3, *Mac1-*ox*-*1-1 and *Pmk1-Mac1-*ox-2-2 (**Figure 3D**). The results indicated that both *Pmk1* and *Mac1* are important for the conidiation of *M. oryzae* strain AM16. In addition, we found that the number of conidia produced by strain *Mac1*-ox-1-1 was more than 30 times that of strain *Pmk1-*ox-9-3 (**Figure 3C**), suggesting that the role of *Mac1* in conidiation was greater than that of *Pmk1*.

### 3.3 Mutations of *Mac1* and *Pmk1* genes significantly impaired conidial germination and functional appressorium formation in strain AM16

**Figure 4:**
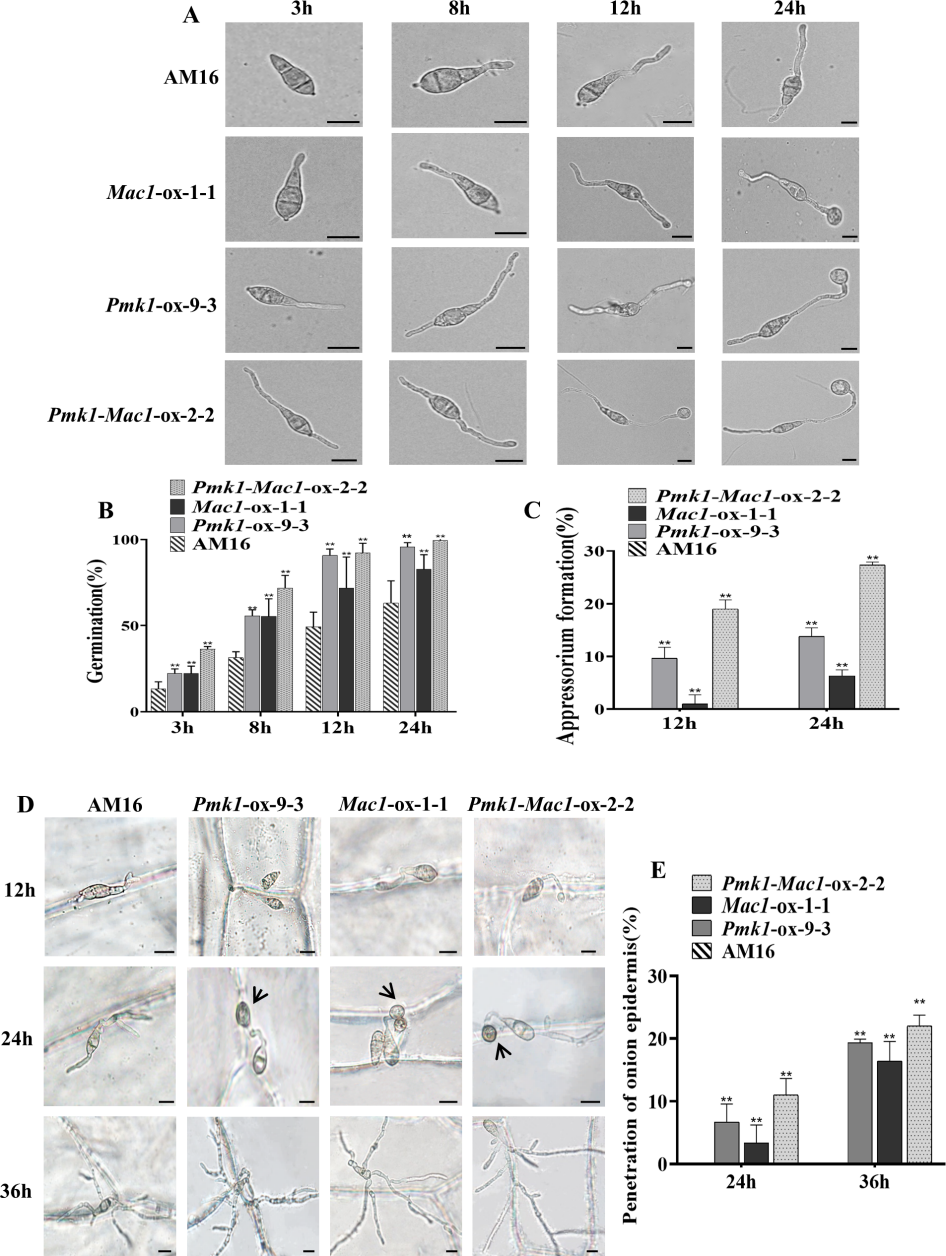
Conidial germination and functional appressorium formation. **(A)** Conidial germination and appressorium formation. **(B)** Conidial germination rate (%) of *P. oryzae* strains at 4, 8, 12, 24 hpi. **(C)** Appressorium formation rate (%) of *P. oryzae* strains at 12 and 24 hpi. **(D)** Conidial germination, appressorium formation and penetration onion epidermis. **(E)** penetration rate (%) of *P. oryzae* appressorium at 24 and 36 hpi. Error bars represent mean ± SD (n=3). Asterisks indicate significant differences from AM16 strain according to Student’s t-test (**p < 0.01). Scale bars, 10 µm.

The formation and maturation of appressorium is one of the key steps for successful plant infection by *M. oryzae*. To clarify the effects of mutations of *Pmk1* and *Mac1* genes on conidial germination and appressorium formation in strain AM16, we performed the investigations of conidial germination and appressorium formation of strains AM16, *Pmk1-*ox-9-3, *Mac1-*ox-1-1 and *Pmk1-Mac1-*ox-2-2 on hydrophobic surface. At three hours, the average percentage of conidial germination in strains AM16, *Pmk1-*ox-9-3, *Mac1-*ox-1-1, and *Pmk1-Mac1-*ox-2-2 was 13.3 ± 4.041%, 22.3 ± 2.517%, 22.3 ± 4.163%, and 36.3 ± 1.528%, respectively, 8 hours, 31.3 ± 3.512%, 55.7 ± 3.512%, 55.3 ± 10.214%, and 71.7 ± 7.506%, respectively (**Figure 4B**). At 12 hours, an average of 9.7 ± 2.082%, 1 ± 1.732%, and 19 ± 1.732% appressorium formation rates were observed in strains *Pmk1-*ox-9-3, *Mac1*-ox-1-1, and *Pmk1-Mac1-*ox-2-2, respectively, 24 hours, 13.8 ± 1.652%, 6.3 ± 1.155%, and 27.3 ± 0.577%, respectively, while no appressorium formation was observed in strain AM16 at the same time point (**Figures 4A, C**). The above results show that *Pmk1* and *Mac1* genes have similar role in conidial germination of *M. oryzae* and with partial functional redundancy. However, the percentage of appressorium formation in *Pmk1*-ox-9-3 much higher than that of *Mac1*-ox-1-1, suggesting that *Pmk1* plays a more important role in appressorium formation. Although strains *Pmk1*-ox-9-3, *Mac1*-ox-1-1 and *Pmk1*-*Mac1*-ox-2-2 could significantly restore the defects in conidial germination and appressorium formation of AM16 strain, not to the level of virulent strain Guy11 with an average of 53.3 ± 2.076% and 96.1 ± 0.741 conidial germination rates at 3 hours and 8 hours, respectively (**Supplementary Figure S7B**), an average of 42.0 ± 2.304% and 64.5 ± 1.903% appressorium formation rates (**Supplementary Figure S7C**). In addition, similar to AM16 strain, we also observed conidial bipolar and even multipolar germination, and excessive growth of germ tube in *Pmk1*-ox-9-3, *Mac1*-ox-1-1 and *Pmk1*-*Mac1*-ox-2-2 (**Figure 4A**; **Supplementary Figure S7A**), suggesting that *Pmk1* and *Mac1* mutations are only part of the cause for the loss of virulence in strain AM16.

To assess the tissue invasion ability of appressoria formed by strains *Pmk1*-ox-9-3, *Mac1*-ox-1-1 and *Pmk1*-*Mac1*-ox-2-2, we conducted the penetration assays of appressoria on onion epidermis. Conidial suspensions (6 x 10^4^ conidia/mL) were inoculated onto the inner epidermis of fresh onions. We observed that the tip of germ tubs in *Pmk1*-ox-9-3, *Mac1*-ox-1-1 and *Pmk1*-*Mac1*-ox-2-2 was beginning to swell at 12 hours (**Figure 4D**). At 24 hours, the average penetration rates were 6.7 ± 2.887%(n=60), 3.3 ± 2.887% (n=60)and 11 ± 2.646%(n=63) in *Pmk1-*ox-9-3, *Mac1-*ox-1-1 and *Pmk1-Mac1-*ox-2-2, respectively (**Figure 4E**). At 36 hours, the average penetration rates were 19.3 ± 0.577%(n=62), 16.4 ± 3.114%(n=61) and 22 ± 1.732%(n=62) in *Pmk1-*ox-9-3, *Mac1-*ox-1-1 and *Pmk1-Mac1*-2-2, respectively (**Figure 4E**). The penetration rate of the strain *Pmk1-Mac1*-ox was similar to the strain *Pmk1*-ox, and which was higher than that of the strain *Mac1*-ox. However, AM16 conidia only formed germ tubes along the cell wall and did not invade the onion epidermis at later stage (**Figure 4D**). These results indicate that mutations of Pmk1 and Mac1 impaired functional appressorium formation in the AM16 strain, and Pmk1 plays a more pivotal role in this process.

### 3.4 Overexpression of *Mac1* ^Guy11^ or (and) *PMK1* ^Guy11^ allele in avirulent strain AM16 restored pathogenicity to rice plants

**Figure 5:**
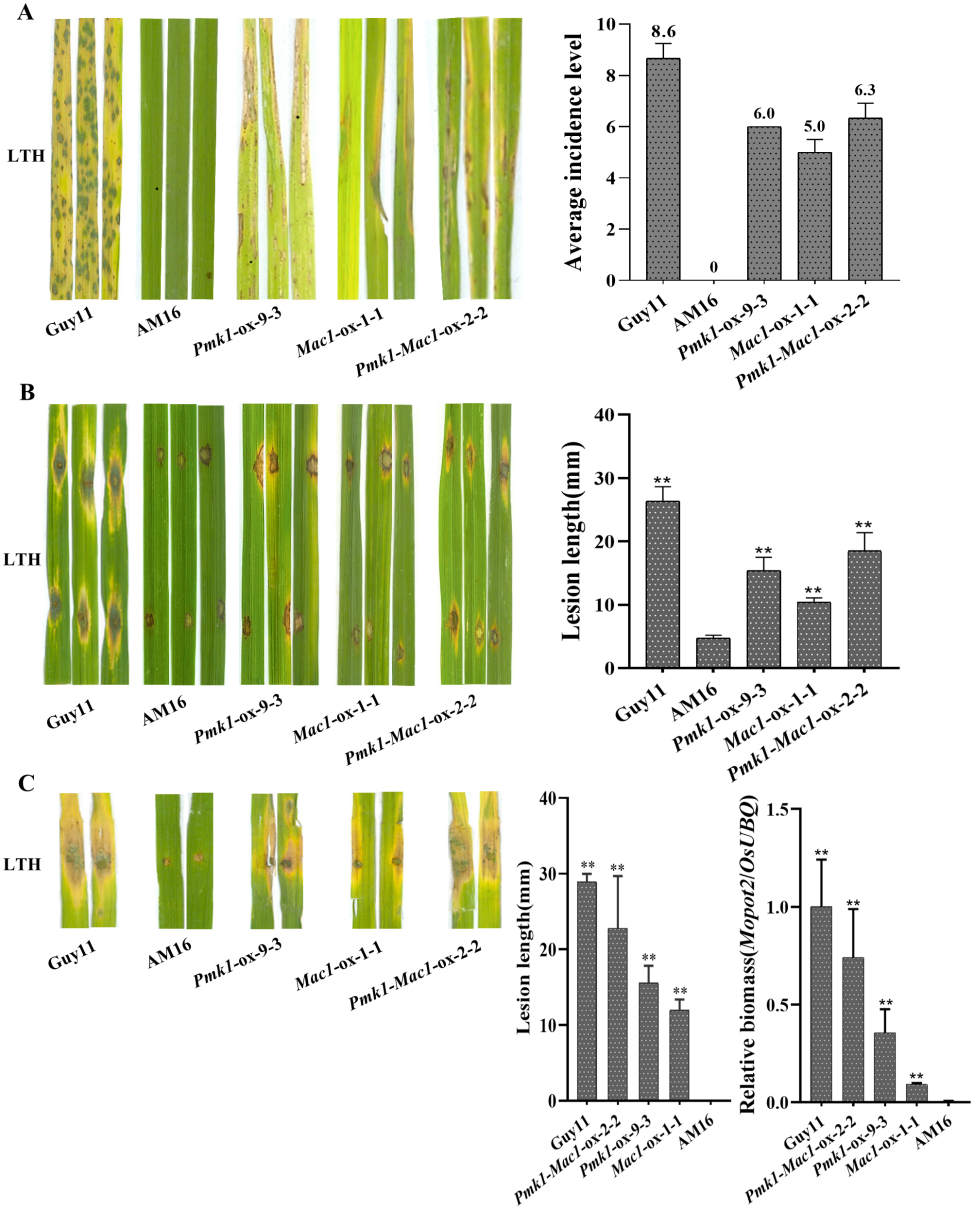
Pathogenicity assays of five *M. oryzae* isolates. **(A)** Rice seedlings of LTH were inoculated with strains Guy11, AM16, *Pmk1*-ox-9-3, *Mac1*-ox-1-1 and *Pmk1*-*Mac1*-ox-2-2 by spraying, respectively. Three inoculated leaves are shown. Disease severity was investigated at 5 dpi. **(B)** Punch inoculation of LTH leaves *in vitro* with strains Guy11, AM16, *Pmk1*-ox-9-3, *Mac1*-ox-1-1 and *Pmk1*-*Mac1*-ox-2-2, respectively. Three inoculated leaves are shown. Lesion length and fungal growth were determined on inoculated leaves at 5 dpi. **(C)** Punch inoculation of LTH leaves *in vivo* with strains Guy11, AM16, *Pmk1*-ox-9-3, *Mac1*-ox-1-1 and *Pmk1*-*Mac1*-ox-2-2, respectively. Two inoculated leaves are shown. Lesion length and fungal growth were determined on inoculated leaves at 5 dpi. The number of biological replicates for each experiment was n=3. Error bars represent mean ± SD (n=3). Asterisks indicate significant differences from AM16 strain according to Student’s t-test (** p < 0.01).

To further understand the mutations of *Pmk1* and *Mac1* involved in the loss of virulence in AM16 strain, LTH seedlings were inoculated with isolates AM16, *Pmk1*-ox-9-3, *Mac1*-ox-1-1 and *Pmk1*-*Mac1*-ox-2-2. The results showed that AM16 was avirulent to LTH same as our previous studies (Jiao et al., 2023), while strains *Pmk1*-ox-9-3, *Mac1*-ox-1-1 and *Pmk1*-*Mac1*-ox-2-2 all are pathogenic to LTH, but the pathogenicity of these strains did not restore to the level of virulent strain Guy11 (**Figure 5**), which was consistent with the results of conidial germination (**Figure 4B**), appressorium formation (**Figure 4C**). Similar results were obtained through punch inoculation of JNXN and Nip plants (**Supplementary Figure S8**). The results indicated that the mutations of Pmk1 and Mac1 are important, but not all, responsible for the loss of virulence in the AM16 strain.

To further demonstrate the correlation between virulence loss of AM16 and the loss of function of Pmk1 protein, we conducted the detection of the expression of Pmk1 protein by Western blot using Anti-MAP Kinase antibody (Clone# MAPK-YT). As shown in **Supplementary Figure S9**, Pmk1 protein was detected in the strains Guy11, *Pmk1-*ox-9-3 and *Pmk1-Mac1-*ox-2-2, but not in AM16(**Supplementary Figure S9**), indicating that the function of Pmk1 protein was indeed lost in the AM16 strain.

### 3.5 Conidiation of AM16 strain was increased in the presence of exogenous cAMP derivatives

**Figure 6:**
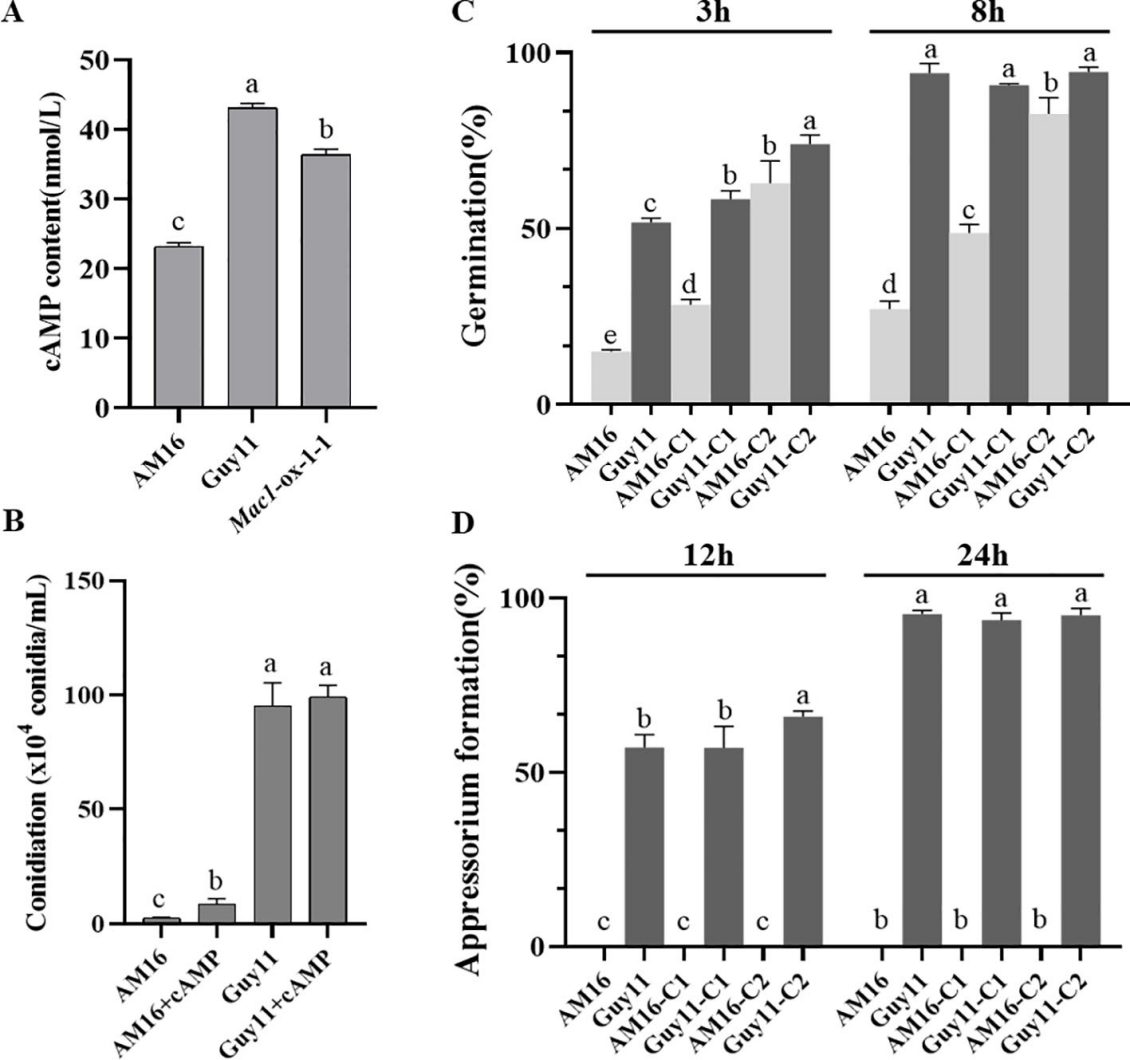
Effect of cAMP on the development of *M. oryzae* AM16 strain. **(A)** Concentrations of cAMP in *M. oryzae* isolates AM16, Guy11 and *Mac1*-ox-1-1. Error bars represent mean ± SD (n=4). **(B)** Conidiation on sporulation medium (OTA plate) containing 5 mM exogenous cAMP. **(C)** Effects of exogenous cAMP on conidial germination. **(D)** Effects of exogenous cAMP on appressorium formation. C1: conidia collected from OTA plate containing 5 mM exogenous cAMP; C2: conidia collected from OTA plate containing without exogenous cAMP, exogenous cAMP was added to conidial suspensions, final concentration 5 mM. For conidial germination and appressorium formation, the concentration of conidial suspension was adjusted to was adjusted to 1 x 10^5^ conidia/mL. Error bars represent mean ± SD (n=3). Different letters above the bars indicate significant differences at p<0.05.

*Mac1* encoding adenylate cyclase, a membrane-bound enzyme that catalyzes the production of cAMP from ATP. Transformants lacking *Mac1* were unable to form appressoria on an inductive surface and were unable to penetrate susceptible rice leaves, but appressorium formation was restored in the presence of exogenous cAMP derivatives (Choi and Dean, 1997). In this study, we found that the transformants of strian AM16 overexpressing *Mac1* ^Guy11^ significantly enhanced the competence of conidiation and appressorium formation (**Figures 3**, **4**; **Supplementary Figure S6**), as well as pathogenicity to rice plants (**Figure 5**; **Supplementary Figure S8**). To determine whether *Mac1* gene mutation also impaired intracellular cAMP accumulation in strain AM16, intracellular cAMP levels of strains AM16, Guy11 and *Mac1*-ox-9-3 were detected by ELISA. The results showed that the intracellular cAMP level of strain AM16 was significantly lower than that of Guy11 and *Mac1*-ox-9-3 (**Figure 6A**), while exogenous addition of cAMP significantly restored the conidiation (**Figure 6B**) and conidial germination AM16 (**Figure 6C**), but did not restore appressorium formation (**Figure 6D**). Although appressorium formation rate of Guy11 was increased at 12 h, there was no significant difference in appressorium formation rate between control and treatment at 24 h (**Figure 6D**). The results showed that *Mac1* gene mutation mainly impaired intracellular cAMP accumulation of AM16, and further reduced the conidiation and conidial germination of *M. oyzae* strain, which is consistent with the higher frequency of the *Mac1* mutation in the natural population of *M. oyzae*.

## 4 Discussion

By analyzing 13 key genes involved in the cyclic AMP, TOR kinase and Pmk1 MAP kinase signaling pathways, we found that the *Pmk1* and *Mac1* genes had specific mutations in the AM16 strain. The studies of the conidiation, functional appressorium formation and pathogenicity to rice of the AM16 strains overexpressing *Pmk1* ^Guy11^ or (and) *Mac1* ^Guy11^ demonstrate that the mutations of *Pmk1* and *Mac1* genes are crucial but not the sole cause of virulence loss in AM16 strain. Both Pmk1 and Mac1 positively regulated conidiation, functional appressorium formation, and pathogenicity to rice in AM16 strain (**Figures 3**–**5**). However, Pmk1 and Mac1 also showed functional divergence, Mac1 plays a more role in conidiation, while Pmk1 has more role in appressorium formation (**Figures 3**, **4**).

Formation of an appressorium by *M. oryzae* requires a conserved pathogenicity mitogen-activated protein kinase (MAPK), called Pmk1. Pmk1 mutants cannot form appressoria or cause plant infection, even when plants are wounded (Xu and Hamer, 1996; Osés-Ruiz et al., 2021; Cruz-Mireles et al., 2024). In the present study, a 29 bp deletion on exon 1 of *Pmk1* in avirulent strain AM16 resulted in premature termination of amino acid translation and loss of Pmk1 protein function (**Figure 1**, **Supplementary Figures S1**, **S9**). Unlike with the above results, the strain *Mac1*-ox overexpressed *Mac1* ^Guy11^in avirulent strain AM16 harboring Pmk1 mutation can form appressoria and cause rice plant infection (**Figure 5**). Upon conidial germination, the key activators and regulators of G-protein/cAMP (Pth11, membrane sensor MoSho1, G-proteins, Rgs1, and Mac1) are internalized to endosomal compartments leading to the accumulation of cAMP and subsequently PKA activity to promote appressorium development and pathogenicity (Fernandez and Orth, 2018). Therefore, we speculate that PKA may be activated by cAMP-dependent signaling in strain *Mac1*-ox to promote appressorium formation and rice plant infection.

*Mac1* encoding adenylate cyclase, a membrane-bound enzyme that catalyzes the production of cAMP from ATP. Transformants lacking *Mac1* were unable to form appressoria on an inductive surface and were unable to penetrate susceptible rice leaves (Choi and Dean, 1997). In this study, The *Mac1* gene in strain AM16 just identified four nucleotide substitution mutations leading to non-synonymous substitutions of three amino acids (T1004K, L1583Q and K2036E) in Mac1, among which T1004K is located in the PLN00113 conserved domain compared to that in strains Guy11 and A7203-8 (**Figure 2**; **Supplementary Figure S3**). Pan-genome analysis revealed that these loci had a certain mutation frequency in the population of *M. oryzae*, and T1004K had the lowest mutation frequency (~20%) among 312 strains analyzed, indicating that unlike the strains lacking *Mac1*, although these mutations could damage the function of Mac1 to a certain extent, reduce the production of conidium, appressorium formation and pathogenicity of the strains, the mutant strains could still infect rice plants and survive in the natural population of *M. oryzae* fungus.

The mechanisms of Pmk1 and Mac1 regulating growth and pathogenicity of *M. oryzae* were studied using knockout mutants or chemical mutants (Mitchell and Dean, 1995; Xu and Hamer, 1996; Choi and Dean, 1997; Adachi and Hamer, 1998; Sakulkoo et al., 2018; Osés-Ruiz et al., 2021). However, the functional differences and connections between Pmk1 and Mac1 in regulating the growth and development of *M. oryzae* remain unclear. The double mutant strain AM16 with Pmk1 and Mac1, a spontaneous mutant, provides an opportunity for us to explore the relationship between Pmk1 and Mac1 in regulating the growth and development of *M. oryzae.* In this study, we first reported that both Pmk1 and Mac1 are necessary for conidiation, appressorium formation, and pathogenicity of *M. oryzae*, but Mac1 plays a stronger role in conidiation than Pmk1, while Pmk1 has more important role in appressorium formation than Mac1 (**Figures 3**, **4**). This discovery provides new information for us to deeply understand the pathogenic mechanism of *M. oryzae*.

AM16 was initially isolated from the diseased samples of rice variety Yixiangyou7633 collected in the field, in 2016. The original strain was pathogenic and identified as physiological race ZE1 (Wang et al., 2022). In 2018, we want to use the strain in our resistance gene cloning study, however we found that it was not pathogenic to three rice varieties (Ziyu44, Jiangnanxiangn, Nippobare) tested in our study. Then we tried to restore the pathogenicity of this strain by growing on complete media for more than 10 generations, but we failed to succeed (unpublished data). Although the pathogenicity of the strain was identified in over 30 rice materials, all rice cultivars tested in our study, including Yixiangyou7633 which used to isolate this strain and Lijiang Xintuan Heigu (LTH, without any *R* gene), were not susceptible to the strain, so we renamed ZE1 to AM (avirulent *M. oryzae*)16 (Wang et al., 2022). AM16 is the only *M. oryzae* strain identified so far that is not pathogenic to the susceptible rice variety LTH, which not only brings a new opportunity for us to deeply study the mechanism of virulence variation of *M. oryzae*, but also a valuable resource for developing inducers of rice immunity. Our study showed that AM16 fermentation liquid had a good effect on the induction of blast resistance in rice (Jiao et al., 2023). Further investigation of the mechanisms underlying the loss of virulence in strain AM16 will provide more molecular evidence for the evolution of virulence in blast fungus.

## Data Availability

The datasets presented in this study can be found in online repositories. The names of the repository/repositories and accession number(s) can be found in the article/**Supplementary Material**.
