# Supplementary material for: Molecular basis for loss of virulence in Magnaporthe oryzae strain AM16
Source: Front Plant Sci. 2024 Dec 6;15:1484214. doi: 10.3389/fpls.2024.1484214 (PMC11659016; doi:10.3389/fpls.2024.1484214)
Supplement: Supplementary file 1 [file DataSheet1.docx]

Supplementary Material

# Supplementary Figures and Tables

## Supplementary Figures


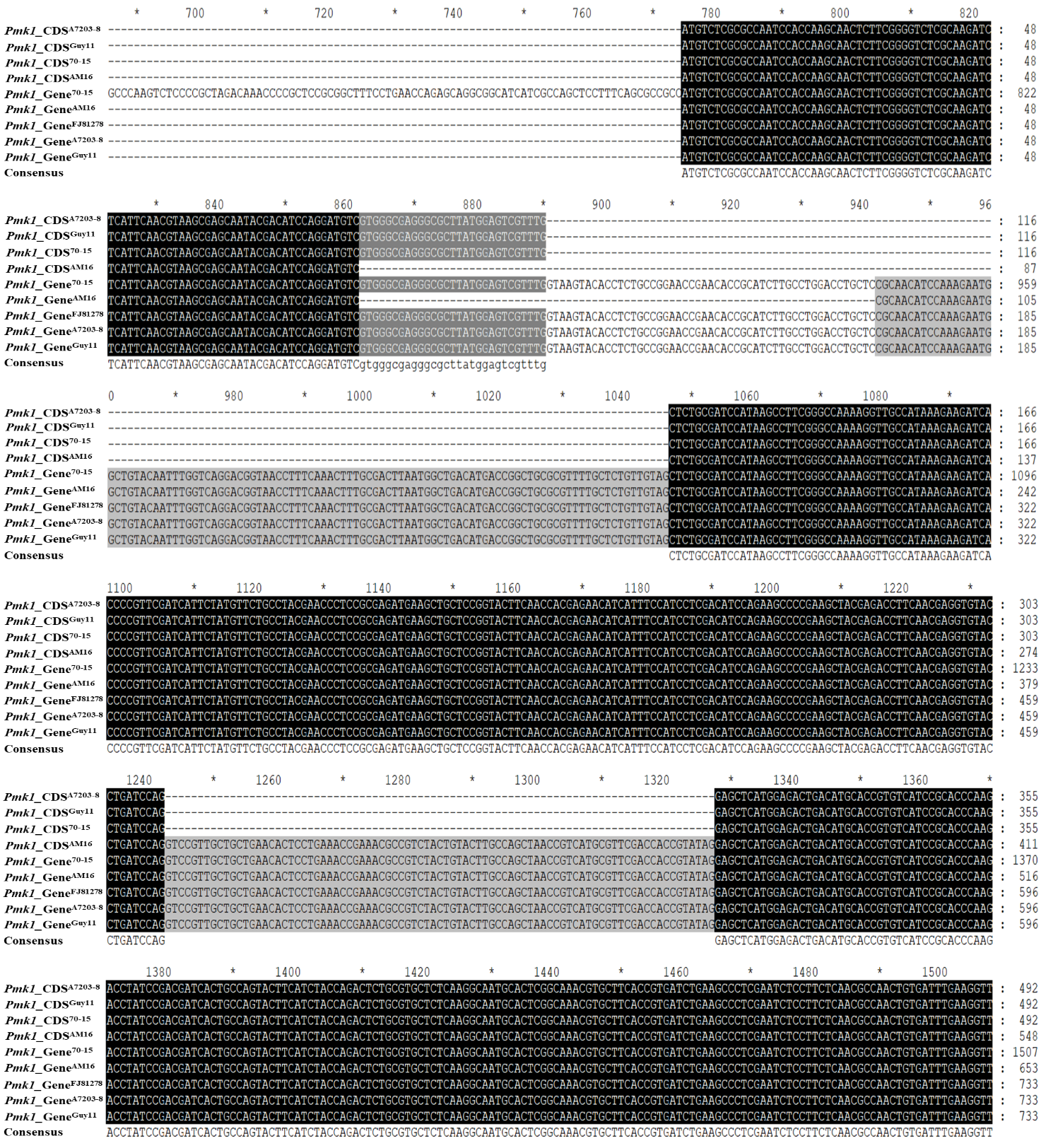


**Supplementary Figure 1.** Alignment of *Pmk1* nucleotide sequences. The CDS sequence of *Pmk1*^70-15^ gene was obtained from NCBI. Gene ID: 2680463. The genome sequence of *Pmk1*^70-15^ was obtained from NCBI. GenBank: GCA_000002495.2. The genome sequences of *Pmk1*^A7203-8^ and *Pmk1*^AM16^ were sequenced by the laboratory itself. The genome sequence of *Pmk1* ^FJ81278^ was obtained from NCBI. GenBank: GCA_002368475.1. The genome sequence of *Pmk1* ^Guy11^ was obtained from NCBI. GenBank: GCA_002368485.1. "^🞲^" represents a separator inserted every 20 bases.


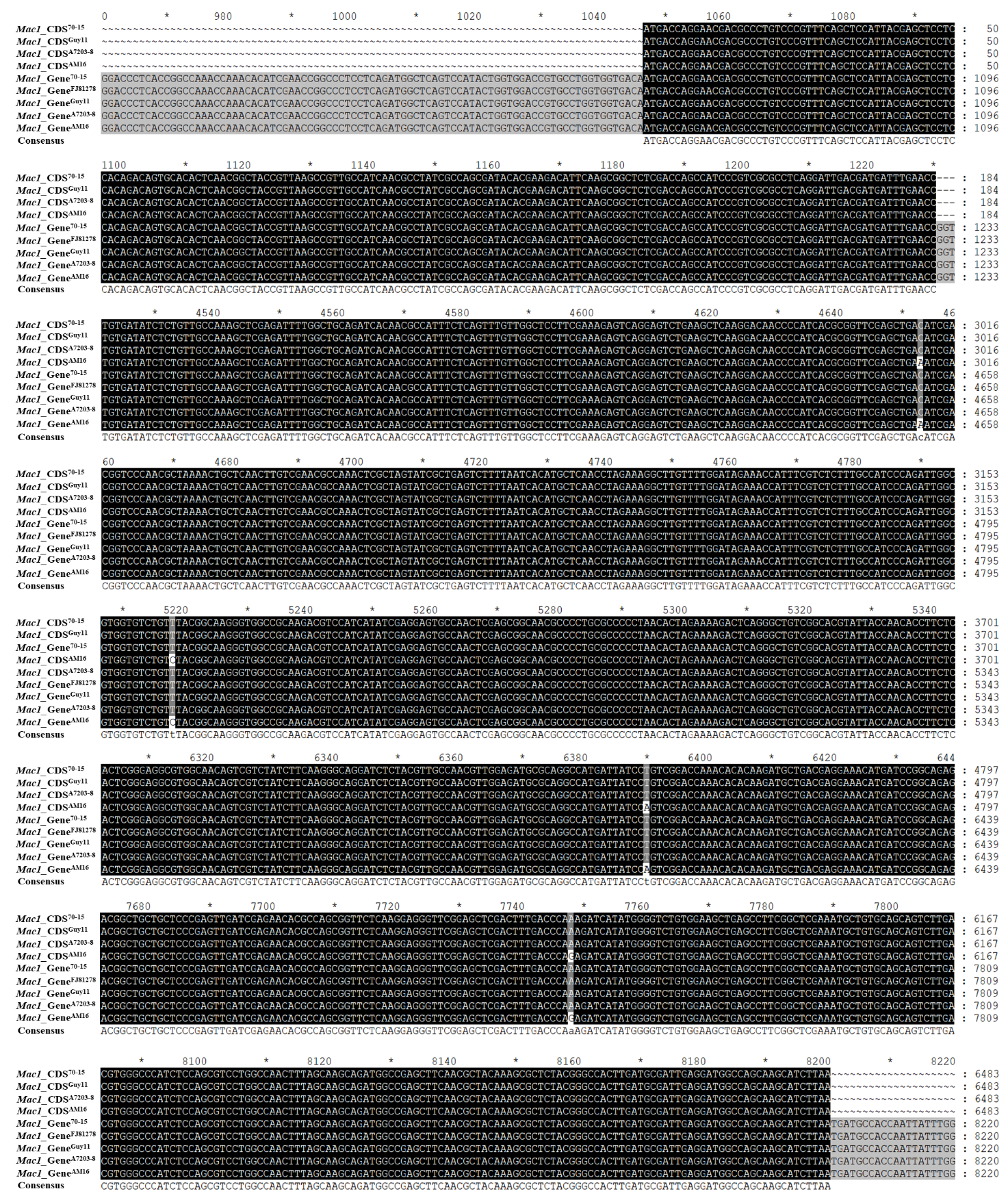


**Supplementary Figure 2.** Alignment of *Mac1* nucleotide sequences. The CDS sequence of *Mac1*^70-15^ gene was obtained from NCBI. Gene ID: 2680463. The genome sequence of *Mac1*^70-15^ was obtained from NCBI. GenBank: GCA_000002495.2. The genome sequences of *Mac1*^A7203-8^ and *Mac1*^AM16^ were sequenced by the laboratory itself. The genome sequence of *Mac1* ^FJ81278^ was obtained from NCBI. GenBank: GCA_002368475.1. The genome sequence of *Mac1* ^Guy11^ was obtained from NCBI. GenBank: GCA_002368485.1. "^🞲^" represents a separator inserted every 20 bases.


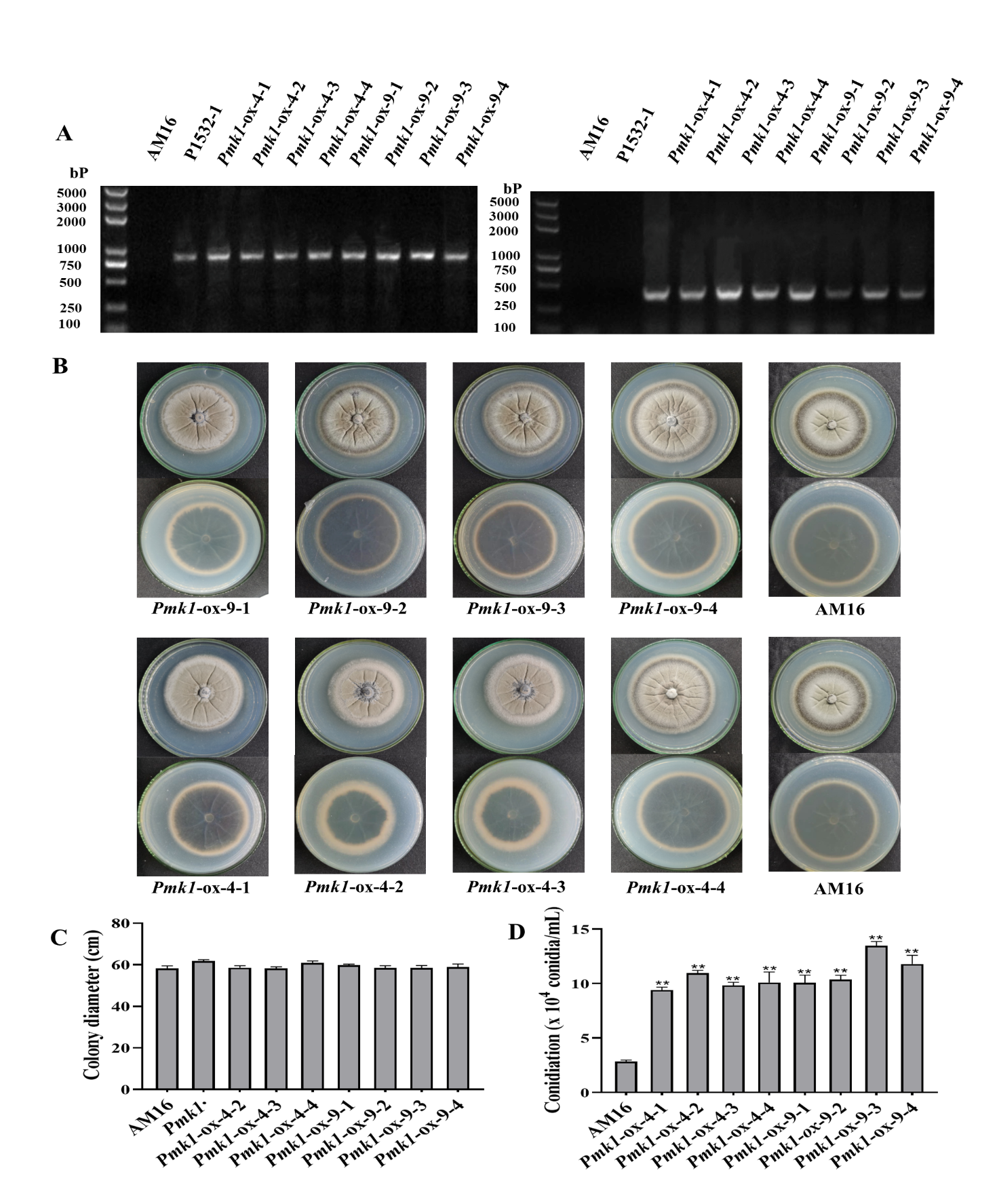
**Supplementary Figure 3.** Identification of positive transformants overexpressing *Pmk1*. (A) Identification of positive transformants. (B) Colony morphology. (C) Colony size. (D) Conidiation. Error bars represent mean ± SD (n=3). "**" above the columns indicates significant differences at P<0.01 with Student’s t-test.


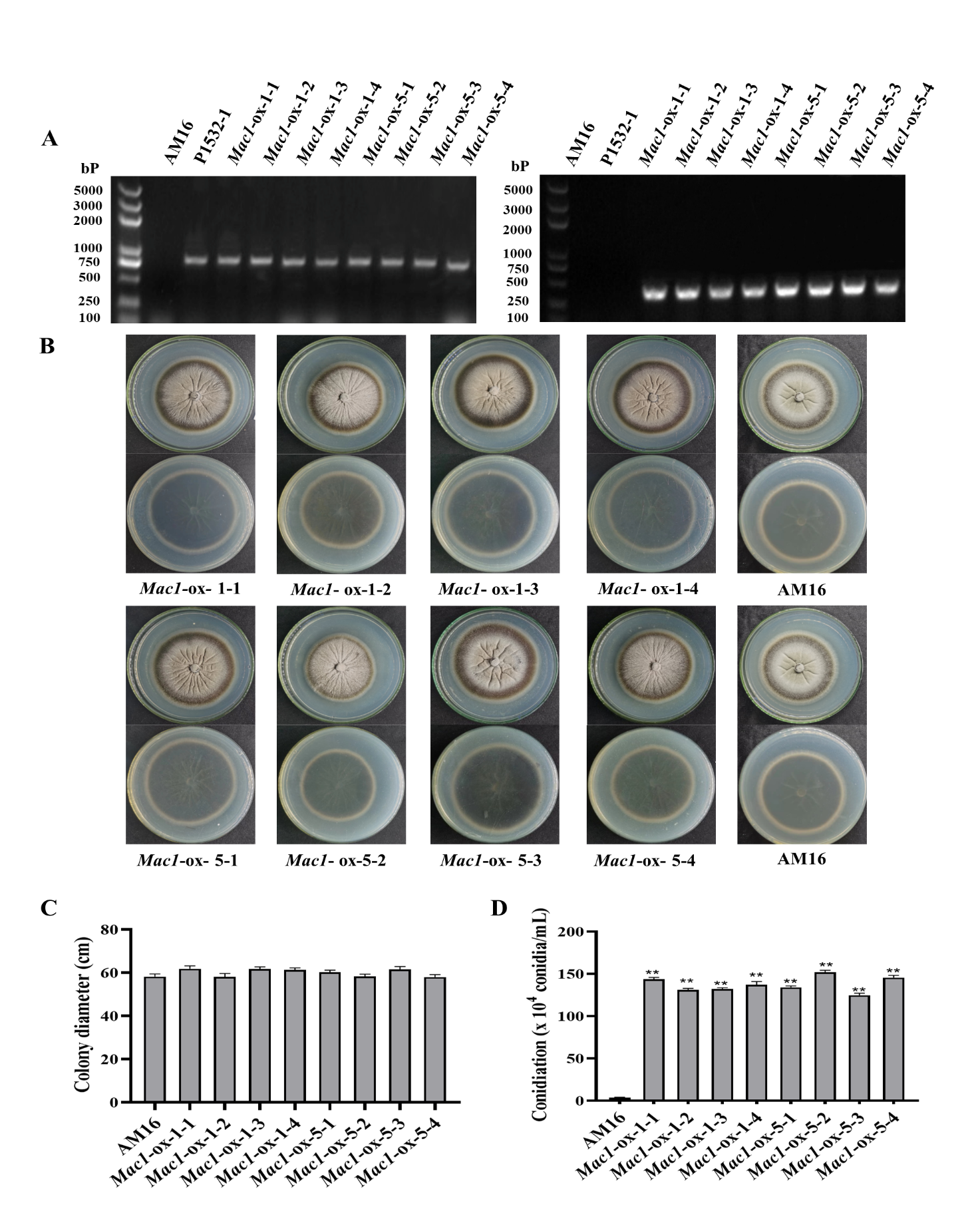
**Supplementary Figure 4.** Identification of positive transformants overexpressing *Mac1*. (A) Identification of positive transformants. (B) Colony morphology. (C) Colony size. (D) Conidiation. Error bars represent mean ± SD (n=3). "**" above the columns indicates significant differences at P<0.01 with Student’s t-test.


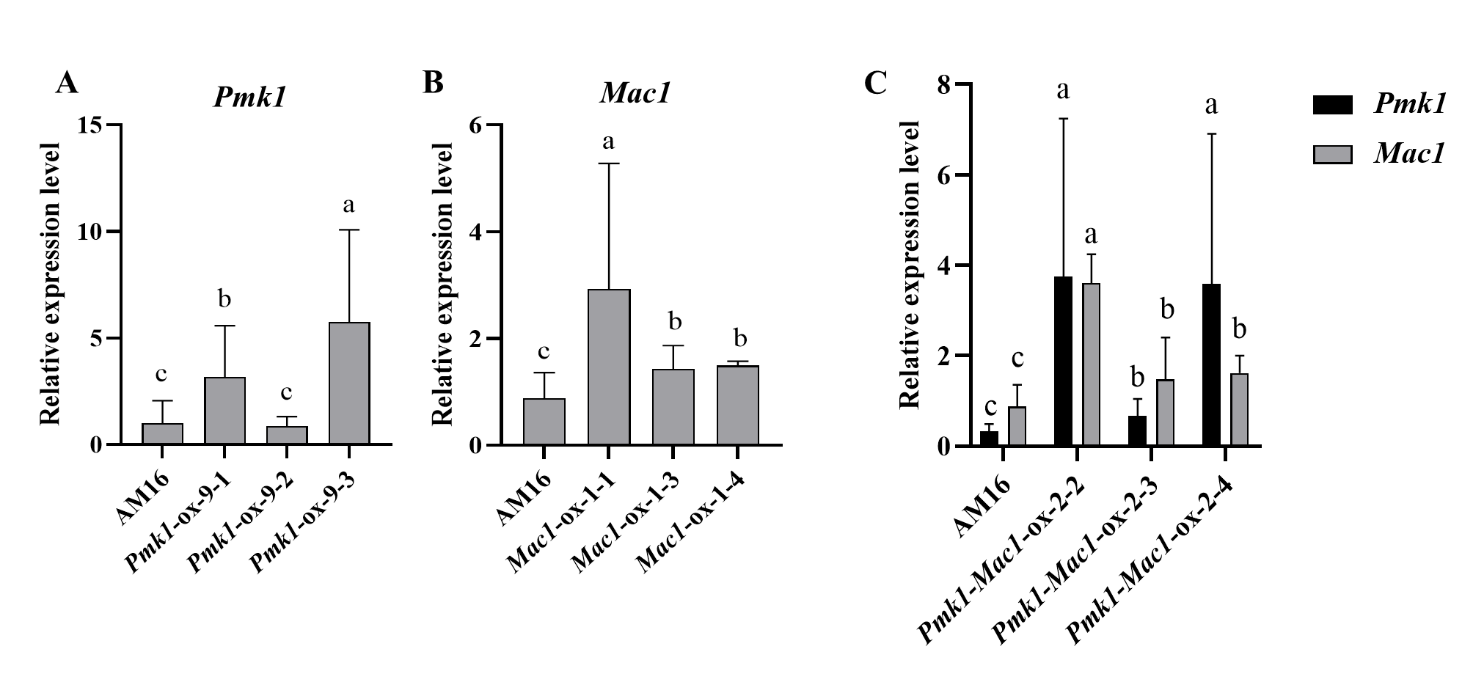
**Supplementary Figure 5.** Expression levels of *Pmk1* and *Mac1* genes in AM16 and its transformed strains. (A) The expression levels of *Pmk1* gene in strains AM16 and *Pmk1*-ox. (B) The expression level of *Mac1* gene in strains AM16 and *Mac1*-ox. (C) The expression level of *Pmk1* and *Mac1* in strains AM16 and *Pmk1*-*Mac1*-ox. The number of biological replicates for each experiment was n=3. Data were analyzed using ANOVA (P < 0.05) and Duncan's test. Different letters indicate the significance at p<0.05. Error bars represent the SD of three replicates (n = 3).


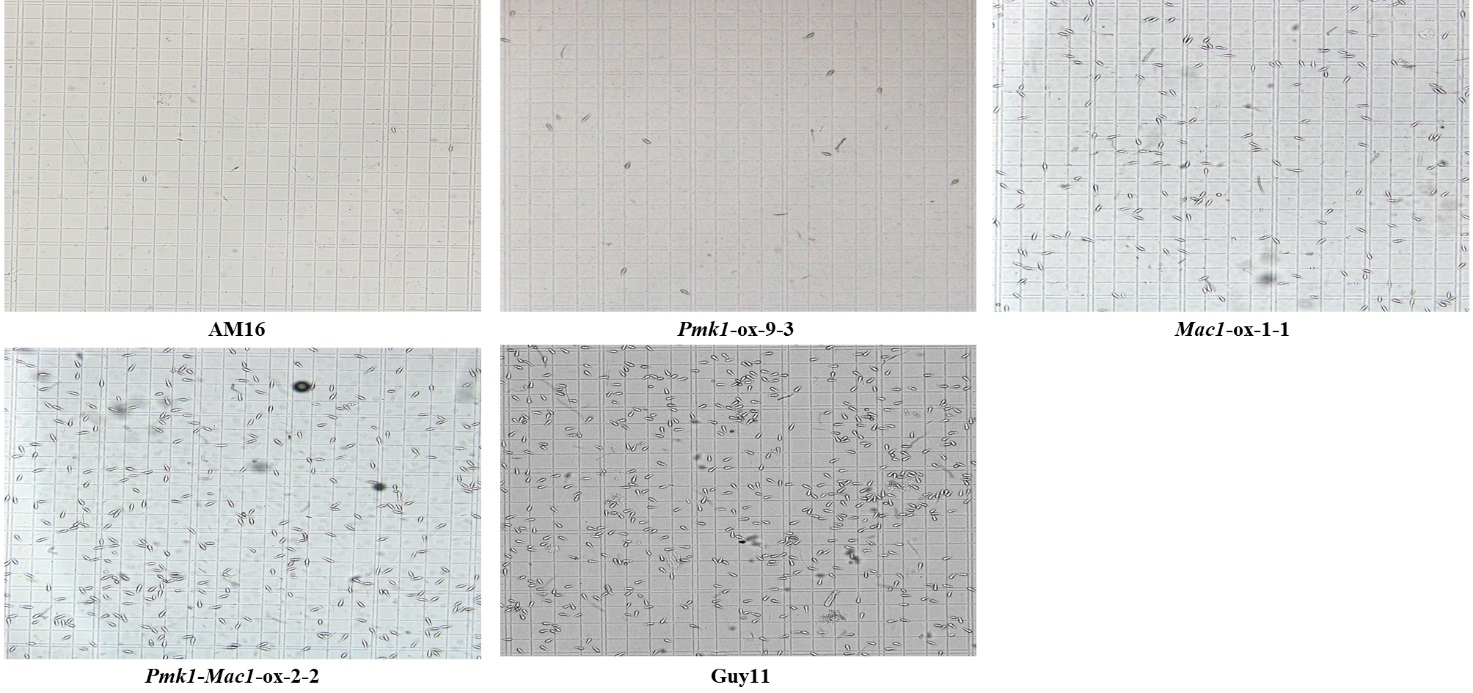


**Supplementary Figure 6.** Conidiation of five *M. oryzae* strains.


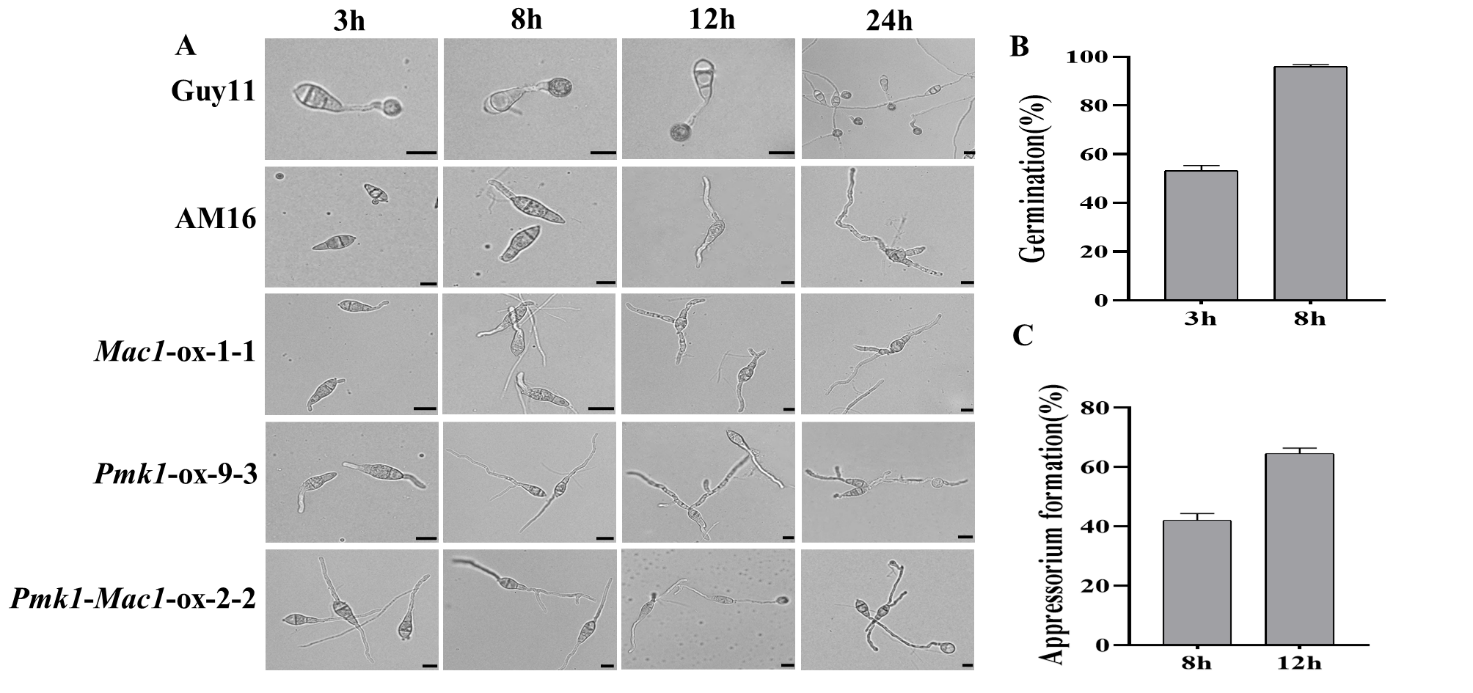


**Supplementary Figure 7.** Conidial germination and appressorium formation on hydrophobic surface. (A) Conidial germination of different strains on hydrophobic surfaces. (B) Conidial Germination rates (%) of Guy11 at 3 hpi and 8 hpi. (C) Appressorium formation rates (%) of Guy11 at 8 hpi and 12 hpi. Error bars represent mean ± SD (n=3). Scale bars, 10 µm.


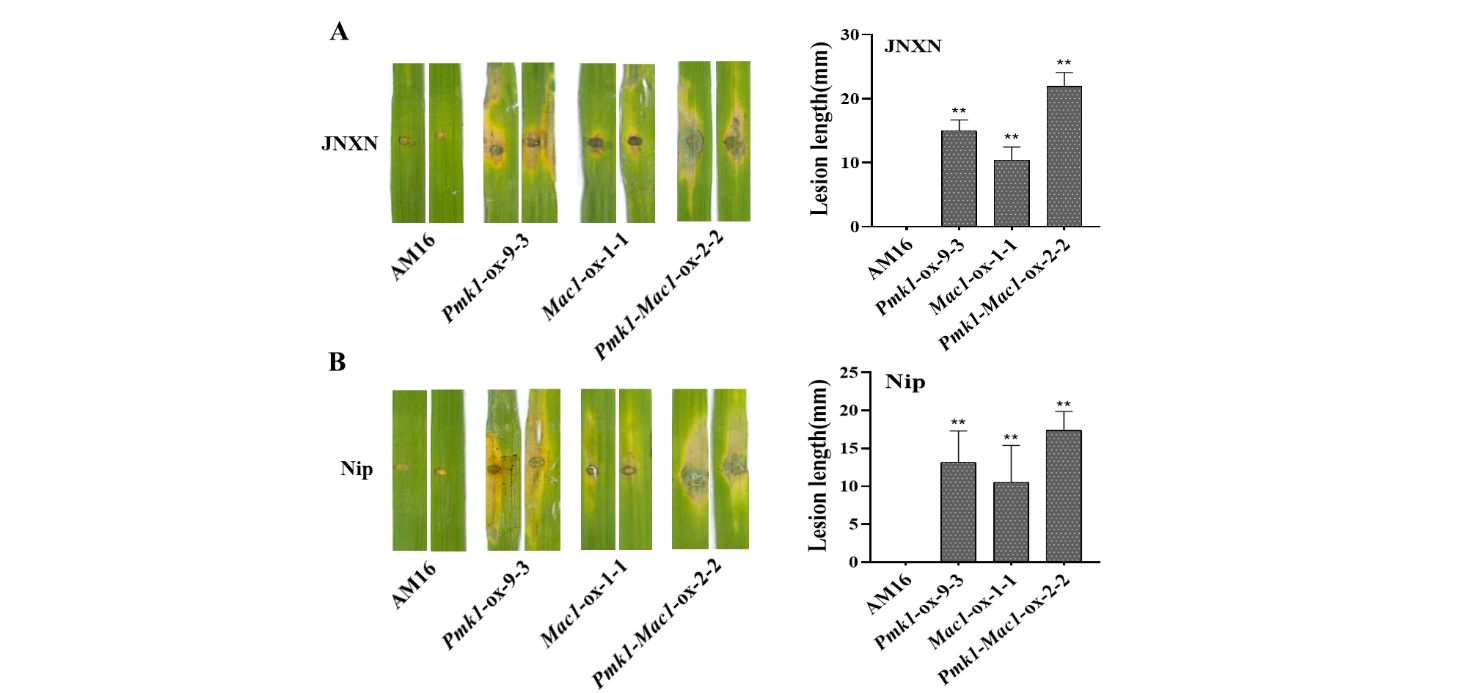


**Supplementary Figure 8.** Pathogenicity of four *M. oryzae* isolates to rice. (A) Punch inoculation of JNXN plants. Two leaves each of AM16, *Pmk1*-ox-9-3, *Mac1*-ox-1-1 and *Pmk1*-*Mac1*-ox-2-2 are shown. Lesion length was measured. (B) Punch inoculation of Nip plants. Two leaves each of AM16, *Pmk1*-ox-9-3, *Mac1*-ox-1-1 and *Pmk1*-*Mac1*-ox-2-2 are shown. Lesion length was measured. Error bars represent mean ± SD (n=3). Asterisks indicate significant differences from AM16 strain according to Student's t-test (** p < 0.01). Jiangnan Xiangnuo (JNXN), Nipponbare (Nip).


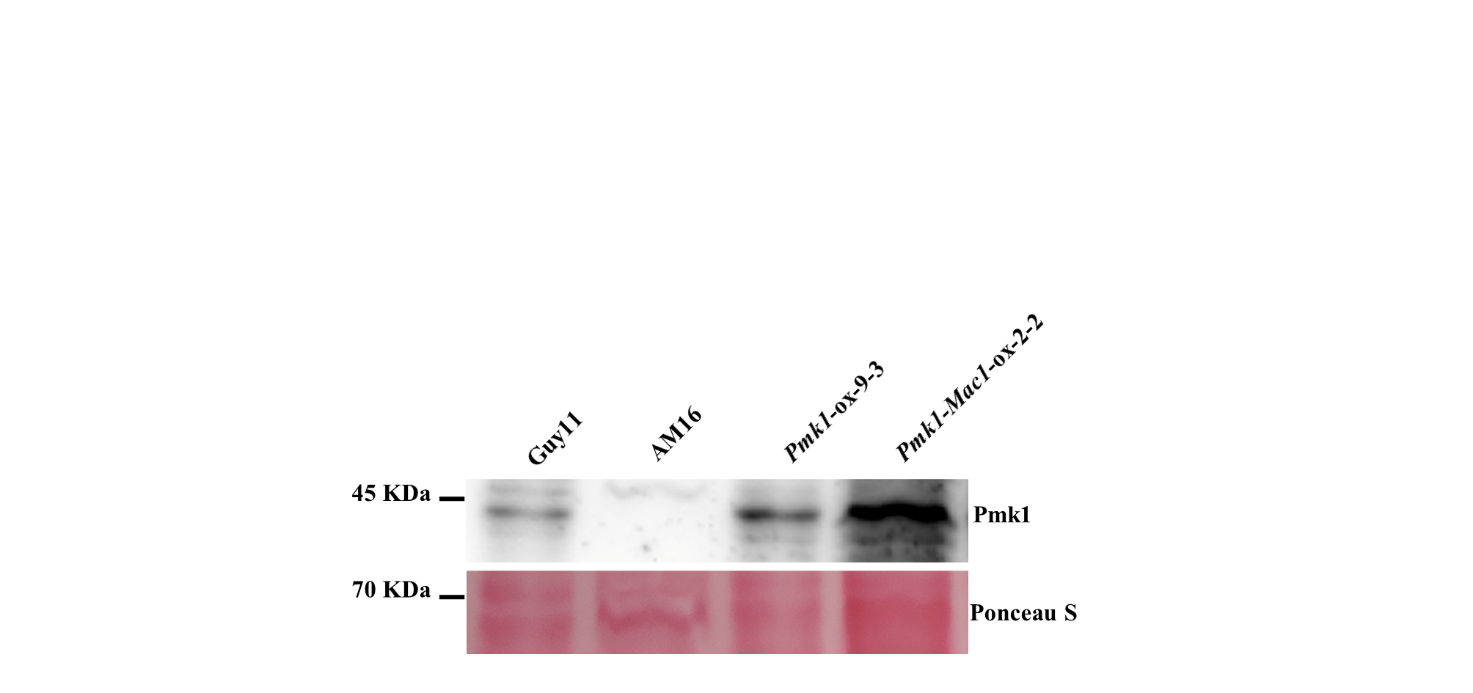


**Supplementary Figure 9.** The expression of Pmk1 protein in different strains. Western blot using anti-Pmk1 antibody (top panel). Ponceau S staining of total proteins was shown as a loading control (lower panel).

## Supplementary Table
